# Trends in complexity of single-agent and combination therapies for solid tumor cancers approved by the US Food and Drug Administration

**DOI:** 10.1093/oncolo/oyae302

**Published:** 2024-11-15

**Authors:** Emerson Y Chen, Manoj Rai, Yash Tadikonda, Preeyam Roy, Dakota W Nollner, Akshit Chitkara, Julia Hamilton, Rajat Thawani

**Affiliations:** Division of Hematology and Medical Oncology, Knight Cancer Institute, Oregon Health & Science University, 3181 SW Sam Jackson Park Road, OC14HO, Portland, OR 97239, United States; Division of Hematology and Medical Oncology, Knight Cancer Institute, Oregon Health & Science University, 3181 SW Sam Jackson Park Road, OC14HO, Portland, OR 97239, United States; Saint Louis University, St. Louis, MO 63103, United States; Division of Hematology and Medical Oncology, Knight Cancer Institute, Oregon Health & Science University, 3181 SW Sam Jackson Park Road, OC14HO, Portland, OR 97239, United States; Department of Internal Medicine, Oregon Health & Science University, Portland, OR 97239, United States; Department of Internal Medicine, University of California at Riverside, Riverside, CA 92521, United States; Department of Internal Medicine, Oregon Health & Science University, Portland, OR 97239, United States; Department of Medicine, Section of Hematology/Oncology, The University of Chicago, Chicago, IL 60637, United States

**Keywords:** FDA approval, solid tumors, cancer drugs, monotherapy, combination agents

## Abstract

**Background:**

Many FDA-approved cancer therapies, whether as a multiagent combination or as a single agent, have demonstrated only modest clinical benefit. To investigate the drug development landscape, this analysis focuses on whether newly approved drugs are added to existing standards as combination therapy or replace a former drug as monotherapy.

**Methods:**

A retrospective analysis of package inserts and corresponding trials for the treatment of nonhematology solid tumor malignancies from January 2011 to December 2023 was conducted to categorize an approval as monotherapy or combination therapy. Drug characteristics, treatment indications, study design, approval history, and efficacy results were compared between the 2 cohorts.

**Results:**

Among the 292 approval entries and 110 drugs, 193 (66.1%) were monotherapies and 99 (33.9%) were combinations. Combinations, when compared with monotherapies, were more frequently approved as regular than accelerated approval (85 [85.9%] vs 132 [68.4%], *P* <.01), in the first-line setting (66 [66.7%] vs 69 [35.8%], *P* <.01), and with overall survival as the criteria (49 [49.5%] vs 40 [20.7%], *P* <.01). Monotherapies were more likely to be novel drugs compared with combinations (80 [41.5%] vs 14 [14.1%] *P* <.01). Monotherapies were more likely to be small molecule targeted agents, while combinations were more likely to be immunotherapies (*P* <.02). There was no difference comparing the time-to-event endpoints and validated clinical benefit scale, but the median response rate of combinations (46%) was higher than monotherapies (34%, *P* <.01).

**Discussion:**

Given that clinical benefit appears limited in combination therapy compared with monotherapy, drug development could focus on simplifying cancer therapies toward patient-centered paradigms.

**Implications for Practice**

In this analysis of FDA-approved drug indications for solid tumors from 2011 to 2023, little difference between monotherapy and combination regimens with respect to both survival outcomes and the European Society of Medical Oncology-Magnitude of Clinical Benefit Scale would support future trials testing experimental regimens with fewer drugs at a time, perhaps in meaningful sequences, rather than combining all available drugs upfront.

## Introduction

The U.S. FDA uses both definite and surrogate endpoints to ascertain the clinical benefit of novel cancer therapies before final approval for commercial clinical use. Over the past 4 decades, over 130 novel molecular entities have been approved for an oncology indication using surrogate endpoints other than overall survival (OS) through regular and accelerated approval pathways.^1,2^ Historically, cytotoxic cancer therapies were commonly validated as monotherapies before being combined into multi-agent regimens to overcome potential therapy resistance. Since then, novel classes of drugs such as small molecule agents targeting specific cell function pathways, monoclonal antibodies targeting hallmarks of cancer receptors, and immunotherapies have become prominent in cancer drug development. In recent years, the increased quantity and complexity of these novel molecular entities have encouraged clinical trials to test multi-agent combination regimens yet again.

Despite the high number of drug approvals by the FDA, many cancer therapies have not met validated frameworks established by oncology societies for meaningful benefit.^3-5^ In total, most cancer therapies may expect to prolong survival by fewer than 3 months.^6,7^ Cancer therapies approved through single-arm studies with low response rates (RRs) are especially unlikely to have long-term benefits.^8^ Positive signals, such as RR and duration of response, from smaller early-phase trials also often do not sustain in larger randomized controlled trials later on.^9,10^ Despite these shortcomings, a number of perhaps mediocre drugs are being increasingly tested in combination therapy trials in the hope of achieving synergistic effects, or at the very least, more obvious responses.

To investigate the changing landscape of combination therapies versus single drug entities in oncology drug registration trials, this analysis focuses on whether newly approved indications for nonhematology solid tumors are added to existing standard as combination therapies or if they are replacing a former inferior standard of care as monotherapy.

## Methods

### Study design

A retrospective review of publicly available package inserts and drug registration trials was conducted to investigate FDA-approved drug therapies for the treatment of nonhematology solid tumor malignancies from January 2011 to December 2023. As a retrospective analysis of publicly available data (https://www.fda.gov/drugs/resources-information-approved-drugs/oncology-cancer-hematologic-malignancies-approval-notifications), local institutional review board submission was not required. However, all research procedures were conducted in accordance with the principles of the Declaration of Helsinki and the best research practices set forth by the local academic institution.

### Data source and collection

All data were sourced from news releases, approval letters, and package inserts publicly available on the US Food and Drug Administration (FDA) website or from published clinical trials available on *PubMed*. Every drug approval was carefully reviewed to extract drug information, drug indications, study design, clinical endpoints, key efficacy results, and approval history. All oncology drugs approved for nonhematologic malignancies from January 1, 2011 to December 31, 2023 were included in this analysis. Indications initially authorized through the accelerated approval pathway, but later, withdrawn remained in this analysis. Drug approvals for precancerous conditions, hematology conditions and malignancies, pediatric cancers, and neoplasms exclusively associated with genetic syndromes were all excluded. Drug entries for topical medications, biosimilars, generics, and minor indication modifications were excluded to prevent duplicates. Likewise, indication modifications based on only longer follow-ups of the same drug registration trial rather than a separate cohort or clinical trial study were also excluded to prevent repetitive entries. All entries were confirmed by at least 2 investigators (M.R., Y.T., A.C., and E.C.) independently, and all data entries were reviewed by at least 2 investigators (M.R., A.C., Y.T., D.N., P.R., and E.C.) to minimize discrepancies. Any reviewer disagreements were resolved through consensus or by consulting a third reviewer when necessary.

### Outcomes

Combination therapy was defined as adding at least 1 experimental drug to an existing standard-of-care regimen or using 2 experimental drugs to form the new cancer therapy indication. For example, adding pembrolizumab (ie, a study drug) to gemcitabine and cisplatin (ie, the standard-of-care chemotherapy backbone) in biliary tract cancers would be counted as combination therapy.^11^ Likewise, the novel combination of enfortumab vedotin-ejfv and pembrolizumab in the first-line treatment of metastatic urothelial cancer is also counted as combination therapy.^12^ In contrast, monotherapy was defined as the use of only one experimental drug to replace an older standard of care. For one, the approval of fruquintinib for colorectal cancer is a monotherapy for treatment-refractory colorectal cancer compared with the best supportive care.^13^ For another, sacituzumab govitecan-hziy is a monotherapy for third-line metastatic breast cancer that replaced other less effective later-line chemotherapies for advanced breast cancer.^14^

### Statistical analysis

Approval pathway type, year of approval, efficacy endpoints, endpoint basis for approval, line of therapy, cancer types, molecular novelty, and drug class were compared between monotherapies versus combination therapies. Efficacy endpoints were broadly categorized into OS, RR, or progression-free survival (PFS), whsich includes other similar composite time-to-event endpoints such as metastasis-free survival and recurrence-free survival. European Society for Medical Oncology Magnitude of Clinical Benefit Scale (ESMO-MCBS) was scored based on validated methods and data already on public website: https://www.esmo.org/guidelines/esmo-mcbs/esmo-mcbs-for-solid-tumours/esmo-mcbs-scorecards. For adjuvant therapy assessments, scores of A, B, and C were assigned 4, 3, and 2 points, respectively. Scores of 4 and 5 are qualified as of substantial benefit. Subgroup analysis of indications in the noncurative metastatic setting was also conducted to minimize missing data. SAS software version 9.4 (SAS Institute Inc.) was used for statistical analyses, with a 2-sided *P* <.05 selected for statistical significance. All tables and figures were constructed using Microsoft Office 2016.

## Results

From 2011 to 2023, 292 unique drug approval entries among 110 molecular entities were identified for nonhematology solid tumor indications. Among them, 217 (74.3%) were regular approvals, and 75 (25.7%) were accelerated approvals (Table 1). Twelve accelerated approvals were later withdrawn. Approved therapy indications were most frequent in the first-line setting (135, 46.2%), among small molecule targeted (116, 39.7%) or immunotherapeutic drugs (107, 36.6%), and during the years 2020-2023 (125, 42.8%). Thoracic cancers (65, 22.3%), dermatologic cancers (35, 12.0%), and breast cancer (33, 11.3%) represented the 3 most common solid tumor cancer indications. Pembrolizumab (41, 14.0%) and nivolumab (27, 9.2%) were the 2 most represented molecular entities.

**Table 1.** General characteristics of cancer therapy indications approved by the FDA from 2011 to 2023.

Characteristic	No. (%) of drug indications (*N* = 292)
FDA approval type	
Regular approval	217 (74.3%)
Accelerated approval	75 (25.7%)
Line of therapy	
Definitive or adjuvant	24 (8.2%)
First-line advanced or metastatic	135 (46.2%)
Second-line advanced or metastatic	106 (36.3%)
Third or later-line advanced or metastatic	27 (9.3%)
Year of approval	
2011–2015	67 (22.9%)
2016–2019	100 (34.3%)
2020–2023	125 (42.8%)
Basis for approval	
RR	99 (33.9%)
PFS	104 (35.6%)
OS	89 (30.5%)
ESMO-MCBS	
One, two, or C	59 (20.2%)
Three or B	113 (38.7%)
Four, five, or A	120 (41.1%)
Study design	
Open-label	192 (65.7%)
Blinded placebo	100 (34.3%)
Control arm	
Former standard therapy	148 (50.7%)
Supportive care or placebo	55 (18.8%)
None (single-arm)	89 (30.5%)
Number of drugs in approved regimen	
One	193 (66.1%)
Two	62 (21.2%)
Three	32 (11.0%)
Four	5 (1.7%)
Study sample size (median, range)	392 (7–5637)

Abbreviations: OS: overall survival; PFS: progression-free survival; RR: response rate.

OS was the primary endpoint and basis for approval in 89 (30.5%) of the indications, while RR endpoint accounted for 99 (33.9%) and PFS endpoint for 104 (35.6%) of the indications (Table 1). With respect to ESMO-MCBS, 59 (20.2%) were scored as 1, 2, or C, 113 (38.7%) as 3 or B, and 120 (41.1%) as 4, 5, or A. Among the 292 drug approvals, 193 (66.1%) were monotherapies, and 99 (33.9%) were combination therapies. Among the combination therapies, 62 had 2 drugs, 32 had 3, and 5 had 4 drugs in the new therapy combinations.

Monotherapies were more likely to be new molecular entities compared with combination therapies (78 of 193, 40.4%, vs 14 of 99, 14.1%, *P* <.01, Table 2). Monotherapies were also more likely to be compared with supportive care, placebo, or no control arm (133 of 193, 68.9% vs 11 of 99, 11.1%, *P* <.01). Monotherapy drugs were more likely to be small molecule targeted agents (86 of 193, 44.6%), while combination therapies were more likely to be immunotherapies (42 of 99, 42.4%, *P* <.02).

**Table 2.** Comparison of approval pathway and scenario between single-agent vs combination cancer therapy indications from 2011 to 2023.

	Monotherapy (*N* = 193)	Combination (*N* = 99)	*P*-value
FDA approval type			
Regular approval	132 (68.4%)	85 (85.9%)	
Accelerated approval	61 (31.6%)	14 (14.1%)	<.01
Line of therapy			
Definitive or adjuvant	17 (8.8%)	7 (7.1%)	
First-line advanced or metastatic	69 (35.8%)	66 (66.7%)	
Second-line advanced or metastatic	84 (43.5%)	22 (22.2%)	
Third or later-line advanced or metastatic	23 (11.9%)	4 (4.0%)	<.01
Basis for approval			
RR	85 (44.1%)	14 (14.1%)	
PFS	68 (35.2%)	36 (36.4%)	
OS	40 (20.7%)	49 (49.5%)	<.01
Control arm			
Former standard therapy	60 (31.1%)	88 (88.9%)	
Supportive care, placebo only, or single-arm	133 (68.9%)	11 (11.1%)	<.01
Study design			
Randomized controlled trials	112 (58.0%)	91 (91.9%)	
Single-arm or multi-cohort trials	81 (42.0%)	8 (8.1%)	<.01
Novelty			
New molecular entity	80 (41.5%)	14 (14.1%)	
Subsequent indication	113 (58.5%)	85 (85.9%)	<.01
Drug class			
Cytotoxic chemotherapy (*N* = 12)	8 (4.1%)	4 (4.0%)	
Immunotherapy (*N* = 107)	65 (33.7%)	42 (42.4%)	
Biologic agents (*N* = 37)	18 (9.3%)	19 (19.2%)	
Small molecular targeted agents (*N* = 116)	86 (44.6%)	30 (30.3%)	
Hormonal (*N* = 14)	10 (5.2%)	4 (4.0%)	
Other (*N* = 6)	6 (3.1%)	0	.02
Sample size (median, range)	275 (7–2840)	612 (23–5637)	<.01

Abbreviations: OS: overall survival; PFS: progression-free survival; RR: response rate.

**Figure 1.**
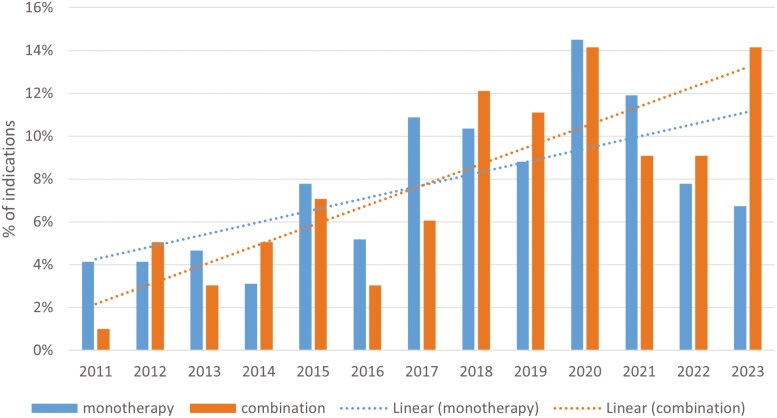
Percentage of drug approvals by year between monotherapy and combination therapies.

**Figure 2.**
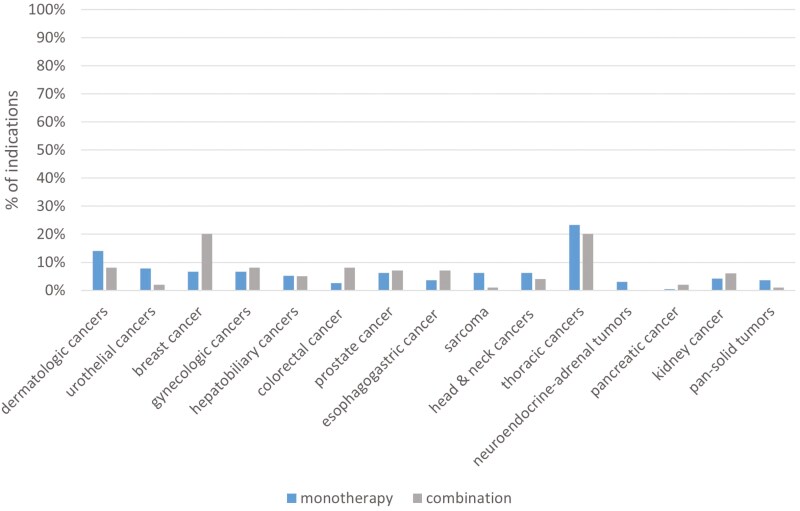
Percentage of drug approvals by cancer type between monotherapy and combination therapies.

Combination therapies were more frequently approved via regular approval than accelerated approval (85 of 99, 85.9%) when compared with single-drug therapies (132 of 193, 68.4%, *P* <.01, Table 2). Combination therapies were more frequently approved in the first-line setting (66 of 99, 66.7%) than monotherapies (69 of 193, 35.8%, *P* <.01). Combination therapies were more likely to have utilized OS endpoint (49 of 99, 49.5%) compared with monotherapies (40 of 193, 20.7%, *P* <.01). RR was more commonly used by monotherapies than combination therapies (85 of 184, 44.1% vs 14 of 97, 14.1%). The indications for both single and combination therapies have similarly increased over the past 13 years (Figure 1). Combination therapies were especially apparent in common cancers, such as lung, breast, and gastrointestinal cancers (Figure 2).

There was no clear difference comparing the PFS and OS endpoints between monotherapies and combination therapies (Table 3). The ESMO-MCBS scores were also similar (monotherapies 73 of 193 [37.8%] vs 47 of 99 [47.4%] scored as substantial benefit, *P* =.28). However, the median RR of combination therapies (46%) was higher than monotherapy (34%, *P* <.01). Sensitivity analysis of only noncurative metastatic indications showed similar results with regards to OS, but there was improved median PFS (8.2 vs 7.0 months, *P* =.02) in addition to the difference in RR endpoint.

**Table 3.** Comparison of magnitude of benefit across endpoints of single-agent vs combination cancer therapy indications approved by the FDA from 2011 to 2023.

	Monotherapy (*N* = 193) Median, range	Combination (*N* = 99) Median, range	*P*-value
Main cohort			
Hazard ratio of OS (*N* = 91; 77)	0.74 (0.36–0.99)	0.74 (0.30–0.95)	.66
Median OS (months, *N* = 77; 68)	18.4 (5.2–81.6)	19.6 (6.1–75.2)	.21
Difference in median OS (months, *N* = 71; 68)	3.9 (−2.80–18.4)	3.6 (−0.60–35.2)	.59
Median PFS (months, *N* = 102; 78)	7.4 (1.7–81.6)	8.2 (2.9–37.2)	.10
Median RR (months, *N* = 164; 89)	34.0% (1.0%–96.7%)	46.0% (5.4%–83.1%)	<.01
Metastatic cohort			
Hazard ratio of OS (*N* = 84; 76)	0.74 (0.36–0.99)	0.74 (0.30–0.95)	.67
Median OS (months, *N* = 77; 68)	18.4 (5.2–81.6)	19.6 (6.1–75.2)	.21
Difference in median OS (months, *N* = 71; 68)	3.9 (−2.80–18.4)	3.6 (−0.60–35.2)	.59
Median PFS (months, *N* = 95; 77)	7.0 (1.7–56.0)	8.2 (2.9–37.2)	.02
Median RR (months, *N* = 164; 85)	34.0% (1.0%–96.7%)	46.0% (5.4%–83.1%)	<.01

Abbreviation: OS: overall survival.

When dividing all approvals to either new molecular entities (ie, first approval) or subsequent approval indications, there was again no clear difference comparing the PFS and OS endpoints between monotherapies and combination therapies (Table 4). While there was an RR difference in subsequent indications (29% vs 46%, *P* <.01), there was no difference in new molecular entities (41.7% vs 38.2%, *P* =.91). A difference in RR was also observed between indications supported by randomized trials versus single-arm studies (median 36% vs 41.7%, *P* <.01).

**Table 4.** Comparison of magnitude of benefit across endpoints of FDA-approved indications of single-agent vs combination cancer therapies within cohorts of new molecular entity and subsequent indications.

	Monotherapy	Combination	*P*-value
New molecular entity (*N* = 94)			
Hazard ratio of OS (*N* = 30; 13)	0.77 (0.36–0.99)	0.77 (0.52–0.95)	.76
Median OS (months, *N* = 28; 12)	16.1 (5.2–81.6)	24.4 (11.5–63.9)	.07
Difference in median OS (months, *N* = 24; 12)	4.0 (−2.80–14.0)	4.7 (−0.30–16.7)	.18
Median PFS (months, *N* = 34; 14)	6.4 (2.0–40.5)	9.6 (3.8–25.3)	.07
Median RR (months, *N* = 75; 14)	41.0% (1.0%–96.7%)	38.2% (18.0%–77%)	.79
Subsequent indication (*N* = 198)			
Hazard ratio of OS (*N* = 61; 64)	0.73 (0.42–0.94)	0.73 (0.30–0.92)	.81
Median OS (months, *N* = 49; 56)	19.1 (5.7–67.0)	18.4 (6.1–75.2)	.57
Difference in median OS (months, *N* = 47; 56)	3.9 (−1.9–18.4)	3.4 (−0.60–35.2)	.81
Median PFS (months, *N* = 68; 64)	7.9 (1.7–81.6)	8.0 (2.9–37.2)	.47
Median RR (months, *N* = 89; 75)	30.0% (2.0–89%)	46.0% (5.4%–83.1%)	<.01

Abbreviation: OS: overall survival.

With respect to the 110 molecular entities representing 292 indications in the analysis, 19 drugs were FDA-approved as a combination regimen first, 10 of which were later approved for other indications still as combination therapy. None were later used as single agent, and 1 was withdrawn. Among the 91 of 110 molecular entities that were approved first as monotherapy, 25 (27%) became approved later as a combination therapy and only 23 (25%) remained as monotherapy in subsequent indications. Seven monotherapy drugs had indications withdrawn or were completely withdrawn from the market.

## Discussion

Combination cancer therapies represented approximately one-third of all drug approvals for nonhematologic solid tumor indications from 2011 to 2023. While combination therapies were more likely to use OS as the primary endpoint and to achieve regular approval, they collectively did not prolong survival duration or improve ESMO-MCBS scores across trials better than monotherapy indications. Treatment response was observed to be higher in combination therapies, but the durable benefit seen in time-to-event endpoints remained unchanged. Several analyses have proposed that the reason for modest survival endpoints, regardless of monotherapy or combination therapies, was due to the use of surrogate endpoints in the initial drug registration trials and approval process.^2,7^ Providing only incremental benefit and supplementing existing therapy regimens may ultimately burden patients by incurring financial and physical toxicities without truly helping them to live longer and better.^15^

Immune checkpoint inhibitors are commonly used with both cytotoxic chemotherapy and targeted agents, as they do not usually have overlapping toxicities with other classes of drugs. Recent studies showed that the proportion of patients who would benefit from immunotherapy is increasing rapidly.^16,17^ Even so, it would still increase the overall side effect profile and costs to the health care system, particularly because most immunotherapy drugs are being tested in combination with cytotoxic chemotherapy or targeted drugs as first-line therapy rather than as maintenance after induction treatment or as replacement therapy for chemotherapy altogether.^18,19^ Notably, when they are approved in the front-line treatment setting, the multi-agent combinations, with their onerous time commitment, cost, and side effects, will impact the largest number of patients. More research could be done to study chemotherapy-free regimens, including more potent or co-formulated immunotherapy drugs, rather than persistently focusing on the chemo-immunotherapy paradigm.

Single-agent therapies, while more common, were more likely to be tested in later lines of therapy, undergo conditional approval through the accelerated approval pathway, and use surrogate endpoints such as RR. Their clinical benefit is sometimes more uncertain, even though some analyses do still support single-arm studies if the experimental drug had high RRs.^20^ Other analyses have echoed the same findings, but they also note that early phase trials and initial drug approvals using surrogate endpoints may have less robust study design and more exaggerated results.^7^ Monotherapies were more likely to be targeted agents, possibly indicating a greater focus on precision medicine and rarer disease cohorts in cancer drug development. The tradeoff here is the allowance of novel drugs into the market for these unique indications with less certainty using smaller and simpler study designs.^21^ In subsequent indications, they may become incorporated in treatment combinations, particularly in the first-line setting, and also achieve regular approval later despite uncertain benefit initially. At this time, up to 68% of drug indications have yet to show survival benefit.^22^ Thus, some monotherapies transition to combination therapies later, up to ~27% in our analysis, thereby propagating the clinical uncertainty to more patients and more disease settings.

Among new molecular entities, there was no clear difference in surrogate or definite endpoint results between monotherapies and combination therapies. This suggests that monotherapy would be a more informative approach to determine the true efficacy of novel cancer drugs. If a more rigorous study design than a single-arm study is applied, more certainty regarding the clinical benefit of a single agent could be established. More development efforts could then be instituted afterward to compare multiple treatment options in a specific sequence rather than combining all of them upfront, and if combination therapies are being planned, OS should be used as the primary endpoint of choice.^23^ Validated frameworks by oncology societies have already been used to differentiate the more promising drugs from the more modest ones.^24^ While there is some biological basis to “use up” all available therapies for aggressive cancer types upfront, there should also be caution in the pharmaceutical incentive environment that prioritizes adding new drugs to existing regimens rather than replacing outdated standards. With combination trials seemingly increasing every year, sometimes with as many as 3 or 4 cancer drugs in 1 combination, more attention should be paid to costs and time to patients, particularly in common cancer types such as lung cancer and breast cancer.

There are several limitations in the study. First, a generalization here is that single-drug therapies are less complex than combination therapies, which may not always be true. Some combination drugs may also be re-formulated as a monotherapy once FDA approved. An alternate analysis could further categorize the drug administration method, overall time commitment, and financial costs to better rate the therapy complexity rather than by the number of drugs. Second, there could be inherent publication bias, as many single-agent and combination therapy trials had negative results and were not approved by the FDA, thereby not included in this analysis. Updated survival data are also not always submitted to the FDA, and long-term survival data may not always be statistically significant. Lastly, there is diversity among cancer drugs, study designs, and drug indications in the list of drug approvals available to the public that is inherently difficult to categorize, but at least, 2 coauthors reviewed most data entries to consistently prepare data for analysis. Further research addressing these issues is encouraged by other investigators in the field.

In conclusion, even in the advent of novel immunotherapy and targeted drugs, cancer therapies for nonhematologic malignancies are increasingly approved by the FDA as combination therapy over monotherapies. While they often utilize definite endpoints such as OS, they do not exhibit better performance in time-to-event endpoints collectively. They also often represent subsequent indications from prior monotherapies that may have utilized surrogate endpoints and less rigorous study designs. Promoting upfront use of multiple cancer drugs concurrently may increase clinical, financial, and time toxicities without improving durable clinical benefit. Patients want to choose unique options based on their personal preferences and situations, not just have 1 single treatment with everything under the sun. Policymakers could encourage drug development to focus on truly innovative drugs over mediocre ones, emphasize rigorous study design to ascertain clinical benefit, and simplify therapy regimens to logical, meaningful sequences rather than consuming them all upfront.

## Data Availability

The data underlying is article are publicly available.
